# A Comprehensive Overview of Subacute Combined Degeneration: MRI Diagnostic Challenges and Treatment Pathways

**DOI:** 10.3390/brainsci15090972

**Published:** 2025-09-10

**Authors:** Caterina Bernetti, Laura Cea, Andrea Buoso, Federico Greco, Mariagrazia Rossi, Fabio Pilato, Rosalinda Calandrelli, Gianfranco Di Gennaro, Vincenzo Di Lazzaro, Bruno Beomonte Zobel, Carlo Augusto Mallio

**Affiliations:** 1 Fondazione Policlinico Universitario Campus Bio-Medico, 00128 Rome, Italy; c.bernetti@policlinicocampus.it (C.B.); laura.cea@policlinicocampus.it (L.C.); andrea.buoso@policlinicocampus.it (A.B.); m.rossi@policlinicocampus.it (M.R.); f.pilato@policlinicocampus.it (F.P.); v.dilazzaro@policlinicocampus.it (V.D.L.); b.zobel@policlinicocampus.it (B.B.Z.); 2 Research Unit of Radiology, Department of Medicine and Surgery, Università Campus Bio-Medico di Roma, 00128 Rome, Italy; federicogreco@outlook.com; 3 Department of Radiology, Cittadella della Salute, Azienda Sanitaria Locale di Lecce, Piazza Filippo Bottazzi, 2, 73100 Lecce, Italy; 4 Unit of Neurology, Fondazione Policlinico Universitario Campus Bio-Medico, 00128 Roma, Italy; 5 Radiology and Neuroradiology Unit, Department of Imaging, Radiation Therapy and Hematology, Università Cattolica del Sacro Cuore, Fondazione Policlinico Universitario Agostino Gemelli—IRCCS, 00168 Roma, Italy; rosalinda.calandrelli@policlinicogemelli.it; 6 Department of Health Sciences, Medical Statistics, University of Catanzaro “Magna Græcia”, 88100 Catanzaro, Italy; gianfranco.digennaro@unicz.it

**Keywords:** neuroscience, neuroimaging, demyelination, myelopathy, axonal damage, vitamin B12 deficiency, neurodegeneration, subacute combined degeneration

## Abstract

Subacute combined degeneration (SCD) is a neurological disorder primarily caused by vitamin B12 deficiency. This condition leads to progressive demyelination and axonal damage, predominantly affecting the dorsal and lateral columns of the spinal cord. This review provides a comprehensive overview of SCD, detailing its complex etiology, pathophysiology, and clinical presentation. We highlight the critical role of magnetic resonance imaging (MRI) in the diagnostic process, discussing both the characteristic spinal cord findings and the more subtle intracranial abnormalities. Furthermore, we address the diagnostic challenges presented by conditions that mimic SCD in MRI, such as multiple sclerosis (MS) and amyotrophic lateral sclerosis (ALS). We conclude by outlining current treatment pathways and identifying key areas for future research, including the use of advanced neuroimaging techniques and the potential for new therapeutic approaches. This updated synthesis aims to provide a clear framework for clinicians and researchers to better understand and manage SCD.

## 1. Introduction

Subacute combined degeneration (SCD) represents a neurological disorder that occurs as a complication of vitamin B12 (cobalamin) deficiency. It involves the spinal cord and manifests as demyelination of the dorsal columns and lateral corticospinal tracts [1].

Vitamin B12 is predominantly acquired through dietary sources, mainly meat, eggs, and dairy products. Absorption of vitamin B12 occurs in the ileum, facilitated by intrinsic factors produced by gastric parietal cells [2].

Factors contributing to vitamin B12 deficiency may include insufficient inadequate dietary intake, compromised B12 absorption due to changes in gastrointestinal structure or function, altered binding or transport of the vitamin, and side effects of certain medications, such as metformin or proton pump inhibitors (PPIs) [2]. Recently, arising between the causes of vitamin B12 dysfunction is chronic N2O recreational use [3,4].

One of the predominant causes of B12 malabsorption remains autoimmune gastritis (pernicious anemia), which triggers the destruction of gastric parietal cells leading to achlorhydria, atrophic gastritis, and reduction of intrinsic factor, which hampers the efficient absorption of vitamin B12 [3,4]. SCD is characterized by multifocal demyelination and axonal damage, predominantly affecting the spinal cord’s white matter. This happens because vitamin B12 plays a pivotal role in various critical biological processes, DNA synthesis, and the maintenance of myelin sheaths around nerve cells, fundamental for neurological functions [3,4]. This condition can manifest with abnormalities of gastrointestinal, hematologic, nervous, and mental systems. From a neuroscience standpoint, SCD is not merely a deficiency syndrome but a clear illustration of the nervous system’s vulnerability to metabolic insults. Understanding the cascade of events—from the disruption of myelin-sustaining biochemical pathways to the resulting axonal injury and functional decline—is crucial for appreciating the intricate relationship between systemic metabolism and neural health. This perspective transforms the view of SCD from a simple hematological-neurological issue to a dynamic process of neurodegeneration. The main reported symptoms in the initial stages of this condition are sensory loss, paresthesia, muscular weakness and ataxia. If left untreated, the condition can progress with more severe consequences such as spasticity and paraplegia. Prompt detection and treatment of vitamin B12 deficiency are vital to avert this severe neurological disorder [5].

In this comprehensive overview we perform a thorough analysis of relevant literature on this particular topic.

### 1.1. Etiology

**Figure 1:**
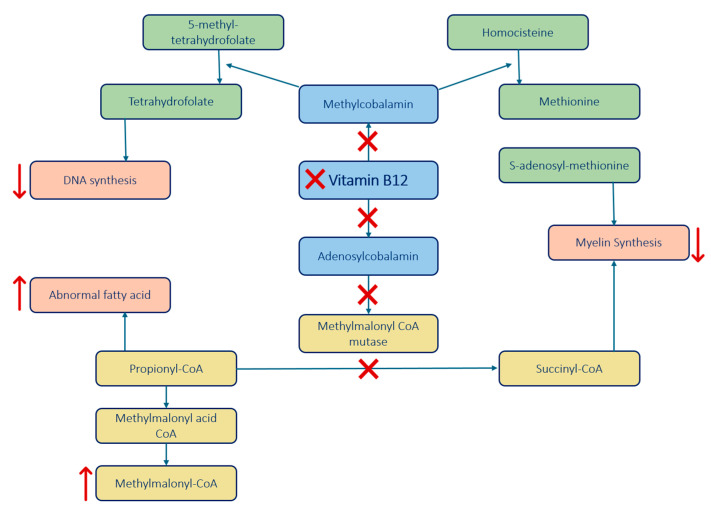
The main enzymes and processes impacted by the lack of vitamin B12, involved in the pathophysiology of SCD. Arrows indicate the reduction or increase of the substance, while crosses represent pathways that are blocked due to the deficiency [1,7].

Vitamin B12 is crucial for DNA synthesis and the metabolism of odd-chain fatty acids, both essential for preserving the integrity of neuronal myelin. To understand the underlying mechanisms of how vitamin B12 deficiency leads to such severe neurological outcomes (Table 1), it is important to explore the specific biochemical pathways affected by this deficiency (Figure 1) [4,6].

Vitamin B12 acts as a cofactor for the following enzymes, both fundamental for myelin formation and preservation [1,7]:

Homocysteine Methyltransferase (enzyme):

- Function: Converts homocysteine to methionine, which is a precursor to S-adenosyl-methionine (SAM) essential for myelin proteins and lipids methylation.
- Impact of B12 Deficiency: B12 deficiency disrupts the conversion, leading to reduced SAM formation, which impairs the methylation process essential for maintaining the myelin sheath.
- Additionally, this enzyme also helps convert 5-methyl-tetrahydrofolate to tetrahydrofolate, crucial for DNA synthesis. B12 deficiency impairs this demethylation impeding effective DNA synthesis.

Methylmalonyl-CoA Mutase (enzyme):

- Function: Converts methylmalonyl-CoA to succinyl-CoA, necessary for myelin synthesis.
- Impact of B12 Deficiency: B12 deficiency leads to an accumulation of methylmalonyl-CoA and propionyl-CoA, which disrupts normal myelin synthesis and leads to the build-up of abnormal fatty acids, contributing to demyelination.
- Vitamin B12 is also involved in cytokine imbalance.
- Findings: Elevated levels of tumor necrosis factor-alpha (TNF-α) and reduced levels of epidermal growth factor (EGF) and interleukin-6 (IL-6) could be involved in the process of demyelination.

Beyond these primary enzymatic roles, the lack of vitamin B12 also leads to the accumulation of homocysteine, a known neurotoxin. Elevated homocysteine levels are implicated in several pathogenic processes, including excitotoxicity, endothelial dysfunction, and oxidative stress, which collectively contribute to neuronal damage and demyelination [8,9]. Furthermore, recent studies have shown that vitamin B12 deficiency can impair mitochondrial function and lead to increased reactive oxygen species, which further compromises the integrity of the myelin sheath and axons through demyelination and axonal damage [10,11,12].

Hence, the notable pathological alterations observed in SCD encompass multifocal, widespread demyelination, and in more severe cases also axonal depletion. These changes primarily occur in the white matter of the dorsal column, lateral corticospinal tracts, and occasionally, the spinothalamic tracts [7].

**Table 1:** Etiological factors of vitamin B12 deficiency leading to subacute combined degeneration (SCD) [1,13,14].

Etiology	Description
Intake deficiency or increased demand	Cobalamin cannot be synthesized by the human body and must be obtained from diet, mainly animal-derived foods. Strict vegetarians and vegans are at a higher risk, especially during periods of increased demand such as pregnancy or lactation.
Gastrointestinal conditions	Stomach: Gastric surgeries (e.g., gastrectomy, bariatric surgery) and conditions such as gastritis or autoimmune gastritis (pernicious anemia), which affect gastric acid and intrinsic factor production, could hamper B12 absorption. Small bowel: Ileal resection, diseases affecting the small intestine (e.g., inflammatory bowel disease—IBD, celiac disease, intestinal motility disorders), bacterial overgrowth, regional enteritis, tropical sprue. These conditions can affect B12 absorption due to reduced absorptive surface or increased bacterial competition.
Pancreatic pathologies	Pancreatic insufficiency or chronic pancreatitis disrupts the cleavage of B12 from proteins, impeding its transfer to the intrinsic factor and reducing absorption.
Drugs	Certain medications can affect B12 availability or absorption mechanisms through various pathways, including long-term suppression of gastric acid (proton pump inhibitors) or interference with calcium-dependent absorption (metformin).
Nitrous oxide (N2O)	Irreversibly oxidizes the cobalt atom in vitamin B12, rendering it inactive. This process inhibits the enzyme methionine synthase, leading to the same metabolic block and neurological consequences as other etiologies. Cessation of N2O use and B12 supplementation are required for neurological recovery.
Genetic anomalies	Neonates may inherit conditions (e.g., transcobalamin deficiency), which affect B12 transport and absorption, leading to early-life deficiencies.

### 1.2. Clinical and Laboratory Findings

It is essential to adequately investigate history, particularly focusing on potential causes of vitamin B12 deficiency, such as dietary habits, which may uncover a nutritional deficiency, previous surgeries, and other pathological conditions, such as malabsorption conditions [15,16,17]. Documentation of alcohol consumption is necessary due to its association with reduced vitamin intake and macrocytosis, but also information about chronic use of medications such as PPI and metformin, as well as sexual history to rule out HIV and neurosyphilis. Inquiries on genetic and autoimmune disorders should also be included [16,18].

A thorough neurological examination is essential; patients with SCD may present with a broad spectrum of symptoms, often evolving gradually over time (Table 2).

Myelopathy presents subacutely in most cases, with a median time from onset to clinical evaluation of 4 months to 1 year, although symptoms can progress rapidly within a few weeks [16].

Early symptoms often include paresthesia and sensory loss with numbness, tingling sensations in the hands or feet, gait ataxia, and muscle weakness in the legs [19]. Distinctive observations also include diminished vibratory senses, heightened reflexes, and spasticity.

Approximately up to 30% of patients with vitamin B12 deficiency experience peripheral neuropathy, which may occur alongside SCD, resulting in myeloneuropathy characterized by both upper and lower motor neuron signs (Table 2) [20]. Persistent vitamin B12 deficiency can lead to cognitive decline and, less frequently, to autonomic and optic neuropathy. As the condition progresses, more severe complications such as gait abnormalities, severe weakness, and even changes in mental status including memory loss or irritability can occur [21,22].

**Table 2:** Neurological symptoms associated with B12 deficiency [1,5,23,24].

Affected Area	Symptoms
Dorsal Columns	Impairment of proprioception, tactile discrimination, vibration sense; tingling, burning, paresthesia in limbs; difficulty maintaining balance without visual cues. Lhermitte’s sign may be present.
Lateral Corticospinal Tracts	Muscle weakness, spasticity, hyperreflexia; initial stiffness, progressing to paraplegia or quadriplegia if untreated; possible sphincter involvement leading to incontinence.
Spinocerebellar Tracts	Gait disturbances, sensory ataxia, positive Romberg’s sign.
Peripheral Nerves and Others	Peripheral neuropathy, visual deficits, neuropsychiatric issues (depression, dementia).

The presence of gastrointestinal symptoms like diarrhea and steatorrhea may indicate malabsorption issues (e.g., inflammatory bowel disease, previous gastrointestinal surgeries) [5,25].

Other symptoms could be anemia-related, such as pallor, fatigue, and signs of congestive heart failure in severe cases. Jaundice may be observed, but also glossitis, which is a relatively common symptom [17,19].

To accurately diagnose SCD specific laboratory tests are essential (Table 3). These tests not only confirm the presence of vitamin B12 deficiency but also help in distinguishing it from similar conditions [20]. The laboratory findings critical for diagnosis are:

Complete Blood Count (CBC) and Blood Smear:

- No anomalies.
- Anemia and macrocytosis: Mean corpuscular volume (MCV) > 115 fL suggestive of B12 vitamin deficiency [15,18,20].
- Hypersegmented neutrophils, mild leukopenia, or thrombocytopenia.

B12 Deficiency:

- Serum B12 Levels: Less than 200 pg/mL; it could be unreliable due to assay variability and only reflects protein-bound B12 [26,27].
- Metabolite Levels: Methylmalonic acid (MMA) and homocysteine levels tested when B12 results are ambiguous [22]. Elevated MMA is more specifically indicative of B12 deficiency, whereas homocysteine can also be elevated in folate deficiency [23].
- Folate Levels: Tested to rule out folate deficiency, which can mimic B12 deficiency signs.
- Anti-intrinsic factor antibodies and serum gastrin levels: Suggestive of pernicious anemia [17,21,25].

**Table 3:** Laboratory findings of SCD [1,7,8,19]. (CBC: complete blood count; MCV: mean corpuscular volume; MMA: methylmalonic acid).

Evaluation Step	Tools and Tests	Key Indicators and Findings
Hematological Abnormalities	CBC, Blood Smear	Anemia Macrocytosis (MCV > 115 fL) Hypersegmented neutrophils
B12 Deficiency Confirmation	Serum B12 Levels, Metabolite Levels (MMA, Homocysteine)	B12 < 200 pg/mL B12 Elevated MMA > 270 nmol/L Elevated homocysteine > 15 μmol/L
Cause of B12 Deficiency	Anti-intrinsic Factor Antibodies, Serum Gastrin Folate	Positive anti-intrinsic factor antibodies, high serum gastrin To rule out folate deficiency

### 1.3. MRI: Key Diagnostic Challenges and Findings

MRI is a critical tool in the diagnosis and evaluation of SCD, providing detailed images that are vital for detecting subtle changes in the spinal cord. MRI findings are crucial for the diagnosis of SCD, with abnormal signals on T2-weighted sequences being present in the majority of cases. While early or very mild cases may have subtle or no findings, most symptomatic patients show characteristic MRI abnormalities in the spinal cord and, less frequently, in the brain. (Table 4) [1].

When clinical symptoms of myelopathy are present, spine MRI including all the spinal cord should be considered, with particular emphasis on utilizing axial and sagittal T2-weighted sequences [28,29,30].

**Figure 2:**
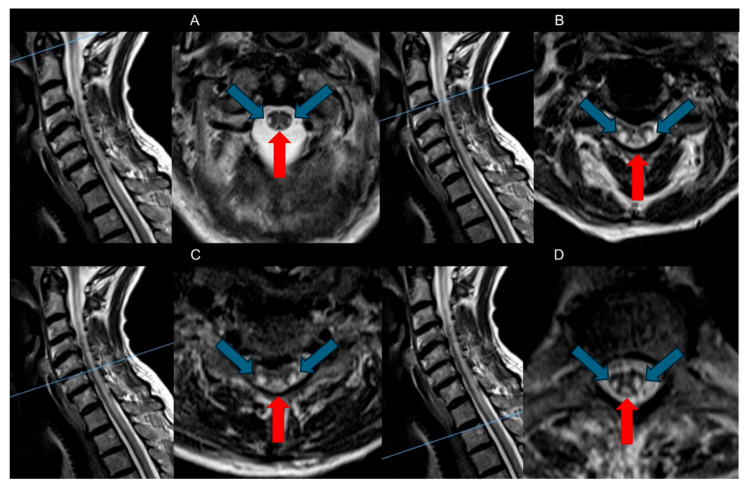
A 74-year-old woman presented with transient facial weakness and speech difficulties, alongside a history of hypertension, diabetes, and cognitive decline. Initial imaging ruled out acute cerebrovascular events, but persistent symptoms prompted further investigation with MRI of the spinal cord showed in the figure. Each image displays a T2-weighted sagittal image on the left, indicating the anatomical level of the lesion, and a corresponding T2-weighted axial image on the right, demonstrating the specific involvement of the spinal cord columns with the characteristic pattern of SCD. MRI revealed bilateral hyperintensities in the cervical and upper thoracic regions, in the dorsal column (red arrows) and the lateral columns (blue arrows), consistent with subacute combined degeneration (SCD) due to severe vitamin B12 deficiency, confirmed by lab tests. The levels shown are: (**A**) Axial view at the C2 vertebral level; (**B**) Axial view at the C3-C4 vertebral level; (**C**) Axial view at the C5-C6 vertebral level and (**D**) Axial view at the T1-T2 vertebral level. Treatment with high-dose vitamin B12 was initiated to prevent further spinal cord damage. (SCD: subacute combined degeneration).

The primary spinal cord changes are demyelination alterations detectable on T2-weighted sequences as bilateral and symmetrical hyperintensities (Figure 2). These changes extend from the upper thoracic regions in an ascending pathway to the cervical tract or descending [31].

- Dorsal Columns: The most characteristic feature observed in SCD is symmetrical bilateral high T2-weighted signal intensities within the dorsal columns of the spinal cord, often referred to as the “Inverted V” sign or “Inverted rabbit ears” sign. This radiological hallmark typically initiates in the upper thoracic region and may show either ascending or descending progression [8,26].
- Lateral Tracts: Usually visible as the disease progresses. Involvement of the lateral corticospinal tracts is more common; less frequently, the lateral spinothalamic tracts are also noted. In rare instances, there can be anterior cord involvement, although this is exceptionally uncommon [1,16,23].

Contrast is not necessary for the diagnosis. Typically, these areas do not exhibit contrast enhancement, although very mild enhancement has been occasionally reported, suggesting a variable response to the inflammatory processes involved in demyelination [15]. The spinal cord caliber is typically normal or slightly enlarged in the acute phase due to edema, while in long-standing, untreated cases, it may appear atrophic [16,32].

While less frequent than spinal cord involvement, brain abnormalities are a recognized feature of SCD. MRI findings typically include symmetrical, diffuse, or patchy T2-weighted and FLAIR hyperintensities in the periventricular and subcortical white matter. In some cases, reversible cerebral atrophy, especially in the frontal and parietal lobes, has been documented, which is correlated with the duration and severity of the deficiency. These findings underscore the widespread impact of vitamin B12 deficiency on the central nervous system, and a comprehensive MRI protocol should include cerebral imaging to fully assess the extent of neurodegeneration and to aid in differential diagnosis [25,33].

Recent literature has expanded on the understanding of these MRI findings, suggesting that the extent and progression of imaging abnormalities could potentially correlate with the severity and duration of B12 deficiency [34,35].

**Table 4:** Summary of key MRI findings in subacute combined degeneration (SCD) [1,25,36].

Spinal Cord		
Dorsal Columns	Bilateral symmetric high signals lesions (inverted “V” sign) in T2-weighted axial sequences	Begins typically in upper thoracic, may progress in the cervical tract Usually no contrast enhancement, rare mild enhancement noted
Lateral Tracts	Involvement possible	
**Brain**		
Cerebral White Matter	Diffuse or patchy T2 hyperintensities	Changes resolve after B12 correction

#### Advanced and Quantitative Imaging

While conventional MRI is the cornerstone of diagnosis, advanced imaging techniques hold promise for providing more detailed insights into the pathophysiology of SCD. Diffusion tensor imaging (DTI), for instance, can quantify microstructural damage to white matter tracts. Reduced fractional anisotropy (FA) and increased mean diffusivity (MD) in the dorsal columns may be used as quantitative biomarkers of axonal loss and demyelination, potentially allowing for the objective monitoring of treatment [37]. Furthermore, the integration of machine learning and artificial intelligence (AI) algorithms could enhance the detection of subtle MRI abnormalities and aid in the differentiation of SCD from other myelopathies, particularly in early or atypical cases [38].

### 1.4. Differential Diagnosis

The differential diagnosis for SCD includes several conditions that affect the dorsal and lateral columns of the spinal cord, presenting similar clinical and radiological findings (Table 5) [16,32].

The main conditions that must be considered are copper deficiency, vitamin E deficiency, or methotrexate-induced myelopathy, because their imaging is indistinguishable from SCD [39]. Hence, in these cases clinical and laboratory findings are essential to obtain the correct diagnosis and to decide the adequate treatment [7,16].

Another important differential diagnosis to consider is with multiple sclerosis (MS). SCD and MS are both demyelinating diseases of the central nervous system and can present with overlapping symptoms such as gait disturbances, sensory deficits, and paresthesias. However, key clinical and radiological distinctions aid in their differentiation. While SCD typically presents with symmetrical paresthesia and posterior column signs, MS is more often associated with relapsing–remitting episodes and a wider array of symptoms, including optic neuritis and internuclear ophthalmoplegia [40]. From an imaging perspective, MRI provides crucial differentiating evidence. The spinal cord lesions in SCD are characteristically confined to the posterior columns, exhibiting a symmetrical “inverted V” or “T2-hyperintense stripe” pattern. In contrast, MS lesions are typically asymmetrical, peripherally located, and often involve multiple tracts, including the lateral and anterior columns [41]. In the brain, SCD can cause diffuse white matter hyperintensities, but these findings are non-specific and usually lack the characteristic periventricular or juxtacortical “Dawson’s fingers” or corpus callosum lesions pathognomonic of MS [41]. Furthermore, unlike SCD, MS is often associated with the presence of oligoclonal bands in the cerebrospinal fluid. Regarding treatment, while vitamin B12 supplementation is curative for SCD, its role in MS is still under investigation. Some studies suggest that B12 supplements may help reduce homocysteine levels and improve quality of life, particularly in patients with co-existing B12 deficiency or elevated homocysteine. However, B12 is not considered a disease-modifying therapy for MS [42].

The differential diagnosis between SCD and ALS can also be challenging due to the overlapping signs of upper and lower motor neuron dysfunction. Both conditions can present with progressive weakness and muscle atrophy. However, a crucial differentiating factor is the presence of sensory symptoms in SCD, which are notably absent in ALS. The progression of SCD is typically slower and reversible with timely treatment, whereas ALS is a relentlessly progressive and fatal neurodegenerative disease. In MRI, the differences are also significant. While SCD shows the characteristic T2 hyperintensity in the posterior and lateral columns of the spinal cord, ALS can show subtle T2-weighted hyperintensities in the corticospinal tracts of the brainstem and internal capsules, reflecting the primary degeneration of upper motor neurons [43,44]. Although vitamin B12 supplementation is the definitive treatment for SCD, its role in ALS is more complex. While it is not a cure, recent studies have investigated the use of ultra-high-dose methylcobalamin to slow disease progression. Post hoc analyses of clinical trials suggest that early administration of methylcobalamin may prolong survival and retard symptomatic decline in certain patient subgroups, possibly by reducing homocysteine levels and promoting neuronal survival, although more research is needed [45,46].

**Table 5:** Differential diagnosis for subacute combined degeneration (CNS: central nervous system; LBSL: leukoencephalopathy with brain stem and spinal cord involvement and lactate elevation; MS: multiple sclerosis; TM: transverse myelitis) [1,16,32,47].

Condition	Spinal Involvement	Distinguishing Features
Nutritional/Metabolic Deficiencies or Toxicity	Dorsal columns, T2 hyperintensity	Low copper/ceruloplasmin Myelodysplastic syndrome Methotrexate use
Demyelinating Disorders: TM and MS [48]	Variable segments may be asymmetric. TM: One or two spinal segments, does not preferentially involve dorsal columns. MS: Asymmetric lesions	Younger age, additional CNS symptoms (MS)
Infectious Causes (Syphilis)	Posterolateral and dorsal columns	Tabe Dorsalis of Syphilis: Lancinating pain, Argyll Robertson pupils
HIV Vacuolar Myelopathy and HTLV-1-Associated Myelopathy	T2-weighted hyperintensity Dorsal columns of thoracic spinal cord Cerebral involvement: More common	Spastic paraparesis Clinical context and specific serological tests (HIV status and CD4 count) are essential: ◯ HIV-VM: Diagnosis of exclusion in patients with advanced HIV infection ◯ HAM: Patients with a history of HTLV-1 infection.
Friedreich’s Ataxia	Dorsal and spinocerebellar tracts	Autosomal recessive (adolescence), hypertrophic cardiomyopathy
LBSL	Entire spinal cord, extends to medulla	Symmetrical involvement, lactate elevation, young age (children)
Other Disorders (e.g., Sarcoidosis, Ischemia, Tumors) [31,32]	Variable	Depending on underlying condition

### 1.5. Prognosis and Complications

Treatment response varies [35]. While approximately 90% of therapies usually stabilize disease progression and lead to clinical improvement, complete resolution is sometimes observed [5]. Prognosis is not influenced by levels of serum vitamin B12 or the severity of anemia. Factors that may indicate a more favorable short-term neurological outcome include being younger than 50 years, a shorter duration of symptoms, absence of sensory deficits, absence of Romberg’s sign and Babinski’s sign, involvement of fewer than seven spinal segments in MRI, presence of spinal cord edema, spinal contrast enhancement, and absence of spinal cord atrophy [49].

If untreated, SCD could lead to severe neurological issues such as paraplegia, quadriplegia, and sphincter incontinence [34]. Persistent anemia may lead to congestive heart failure. Additionally, patients with autoimmune gastritis are at higher risk of gastric carcinoma, carcinoid tumors, and other autoimmune conditions (e.g., diabetes, Hashimoto thyroiditis) [50,51].

### 1.6. Treatment and Management

Vitamin B12 supplementation represents the primary treatment for SCD. The choice of the dosage, route (intramuscular, deep subcutaneous, oral, or sublingual), and duration of vitamin B12 therapy is guided by the severity of symptoms, urgency of treatment, underlying causes, and patient preferences [16,52].

Usually, symptoms improvement after treatment correlates inversely with both their duration and severity of SCD. This connection highlights the critical significance of early diagnosis [53].

For typical cobalamin deficiencies, an oral dose of 1000 mcg daily is usual, or higher in case of malabsorption issues. Meanwhile, in cases of SCD, where rapid intervention is critical to prevent permanent neurological damage, parenteral administration is preferred initially with a recommended regimen of intramuscular cyanocobalamin (1000 mcg) once a week for one month, then monthly. For acute neurological symptoms, an intensive course of 1000 mcg every other day for two weeks is suggested, followed by monthly injections (Table 6). While cyanocobalamin and methylcobalamin are the most common forms of replacement, hydroxocobalamin is also widely used in certain parts of the world and is equally effective in reversing neurological symptoms [54].

Moreover, it is critically important to initiate vitamin B12 supplementation before or concurrently with folate administration. While folate can correct the associated megaloblastic anemia, it can also precipitate or worsen the neurological manifestations of SCD if the underlying B12 deficiency is not simultaneously addressed. High-dose B12 supplementation should be the priority in all cases with neurological signs.

### 1.7. Monitoring and Response to Therapy

**Figure 3:**
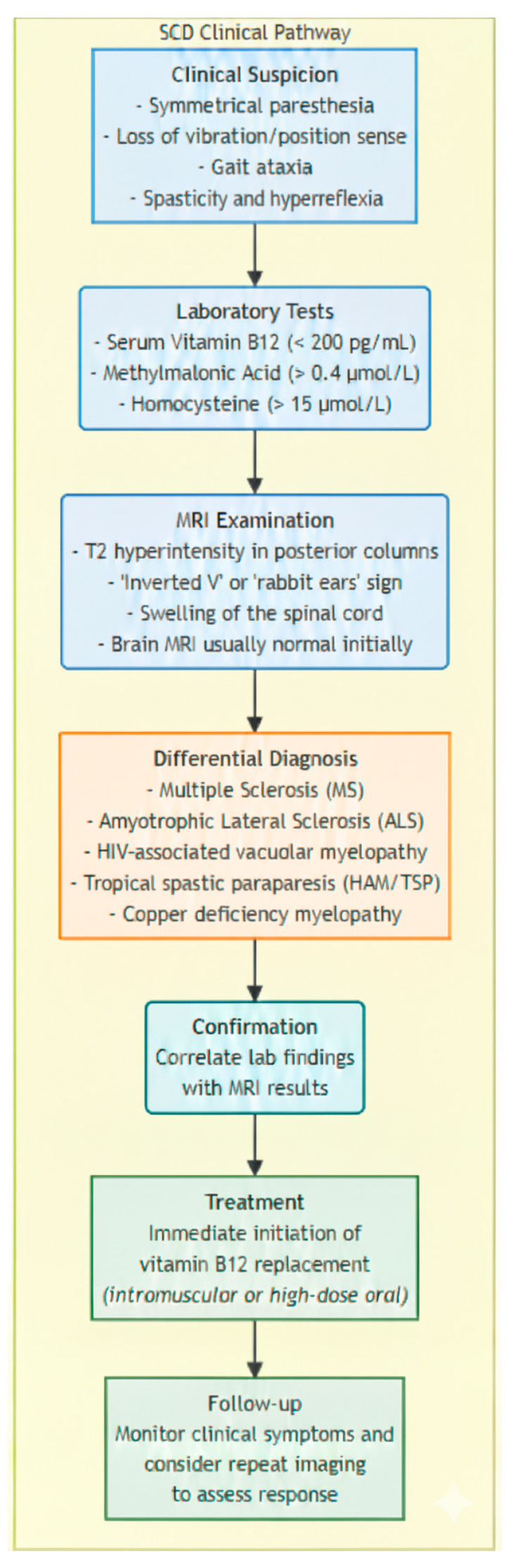
Pathway summarizing the approach to diagnosing and treating SCD.

Patients with irreversible causes such as pernicious anemia require lifelong supplementation, whereas those with reversible causes such as dietary gaps or drug-induced deficiencies may discontinue treatment once levels normalize [35]. If treatment does not yield results, it may be necessary to consider the patient’s adherence to the treatment plan, investigate possible malabsorption issues, or reevaluate the diagnosis to rule out other potential causes of anemia [21] (Figure 3).

The effectiveness of treatment is assessed through improvements in both laboratory values and symptoms:

- It is essential to routinely check vitamin B12 levels, particularly in patients with SCD, and continue monitoring until recovery is complete.
- Markers of hemolysis often begin to decrease within a few days, and reticulocyte counts generally increase within the first week. Normalization of hematological parameters—including anemia, neutrophil segmentation, leukopenia, and thrombocytopenia—is typically observed within two to four weeks [55].
- Additionally, it is crucial to monitor for hypokalemia during the initial treatment phases due to the increased uptake of potassium by cells.
- Neurological impairments, including spinal and cerebral alterations, often improve after correcting vitamin B12 deficiency but may take longer (three to twelve months), highlighting the potential for reversing some neurological damage with timely treatment. However, in more severe cases, the neurological deficits may become permanent [21].

## 2. Conclusions

This article underscores the pivotal role of radiologists in the multidisciplinary management of SCD. Beyond a mere diagnostic tool, timely and targeted imaging is critical in unraveling the underlying pathophysiology of neurological symptoms in complicated cases, leading to more effective and targeted therapeutic interventions. Radiologists must remain vigilant in recognizing the characteristic MRI features of SCD and collaborate closely with neurologists and primary care physicians to ensure a comprehensive approach to patient care. Future research should focus on a more detailed analysis of the prognostic value of MRI features, the potential use of advanced imaging techniques like diffusion tensor imaging to quantify axonal damage, and the application of artificial intelligence and machine learning algorithms to enhance the early detection and monitoring of SCD from routine spinal MRI scans.

## Figures and Tables

**Table 6:** Treatment regimen for subacute combined degeneration (SCD) [7].

Treatment Aspect	Recommendations
Initial Treatment Route	Parenteral (intramuscular) preferred for severe cases
Oral Dosing	1000–2000 mcg daily depending on absorption status
Parenteral Dosing	1000 mcg weekly for one month, then monthly
Treatment Duration	Lifelong for irreversible causes; until correction for reversible causes
Monitoring Frequency	Until recovery
Outcome Expectations	Hematological improvement within weeks; neurological over months
